# Oral Manifestations of Type II Diabetes Mellitus and Comparison of Blood and Salivary Glucose Levels

**DOI:** 10.7759/cureus.42344

**Published:** 2023-07-23

**Authors:** Arun Prasath Alagiriswamy, Meena Gayathry Nagaraj, Karthik Rajaram Mohan, Mohan Narayanan, Priyadeepalakshmi Karunakaran

**Affiliations:** 1 Oral Medicine and Radiology, Sri Ramakrishna Dental College and Hospital, Coimbatore, IND; 2 Dentistry, NG Hospital & Research Centre, Coimbatore, IND; 3 Oral Medicine and Radiology, Vinayaka Mission's Sankarachariyar Dental College, Vinayaka Mission's Research Foundation (Deemed to be University), Salem, IND; 4 Public Health Dentistry, Vinayaka Mission's Sankarachariyar Dental College, Vinayaka Mission's Research Foundation (Deemed to be University), Salem, IND

**Keywords:** blood glucose, xerostomia, dental caries, glycosylated hemoglobin (hba1c), diabetes mellitus type 2

## Abstract

Background and introduction

Diabetes mellitus is a common systemic disease in nearly all countries. Its prevalence has been increasing. Thus, early detection and control of this disorder are urgent tasks. The average blood glucose, salivary glucose, and glycosylated hemoglobin (HbA1c) levels must be estimated.

Aim and objective

This study aimed to assess oral manifestations of type 2 diabetes mellitus in relation to HbA1c and compare glucose levels in the saliva and blood.

Materials and methods

This study examined 60 patients with diabetes who were visiting the Department of Oral Medicine and Radiology as well as the Department of Medicine at Vinayaka Mission’s Sankarachariyar Dental College, Salem, Tamil Nadu, India. In all patients, the blood glucose, saliva glucose, and HbA1c levels were estimated.

Results and statistical analysis

The patients (aged 40-60 years) have type 2 diabetes mellitus; among them, patients aged 56-60 years were predominant. In the Pearson correlation analysis, a positive correlation was obtained in random blood glucose, salivary glucose, and HbA1c levels. The p-value was 0.001**, which indicated significance.

Conclusion

This study revealed a clear-cut correlation between blood and salivary glucose levels. Thus, salivary glucose levels can very well become a substitute for blood glucose levels. If the estimated salivary glucose level is used in practice to estimate glucose levels in patients with diabetes, the use of any invasive procedures may be avoided.

## Introduction

Diabetes mellitus (DM) is the collective term for heterogeneous metabolic disorders whose main finding is chronic hyperglycemia. The cause is either a disturbed insulin secretion or a disturbed insulin effect or usually both [1]. This high glucose level is a mere outcome of decreased insulin secretion or increased cellular resistance to insulin. This condition leads to multiple abnormalities in the metabolism of carbohydrates, fats, and proteins [2]. The World Health Organization’s global status report pertaining to noncommunicable diseases states that “Diabetes is a chronic disease, which occurs when the pancreas does not produce enough insulin or the body cannot effectively use the insulin it produces. This leads to increased concentration of glucose in the blood” [3]. Generally, DM is classified into two types: type 1 DM (DM1, also called insulin-dependent DM [IDDM] or juvenile diabetes) and type 2 DM (DM2, non-IDDM) [4]. Both DM1 and DM2 present numerous possible long-term complications [4,5]. Globally, DM is considered an upcoming looming health issue, constituting a huge public health burden that is predicted to affect 300 million people by 2025 and at least 366 million people by 2030 [6]. Many laboratory and diagnostic procedures are available for the diagnosis of DM, which all revolves around cellular and chemical constituents of the blood and saliva [7]. In this study, blood samples were obtained, and their glycosylated hemoglobin (HbA1c) values were measured. Blood HbA1c level provides evidence on the average blood glucose level of an individual for the previous two to three months and whether a patient with known DM and receiving anti-DM medication has good control over his blood glucose levels. Thus, DM2 is now monitored or assessed using the HbA1c value [8]. To detect blood glucose values, blood samples can be obtained through various methods such as venipuncture, finger stick, and other invasive techniques. Thus, blood collection is always associated with physical and mental stress and pain. To avoid these patient discomforts, the use of saliva as an alternative for the determination of glucose levels was considered to avoid the above-mentioned invasive procedures [9]. Thus, this study examined the correlation between salivary and blood glucose levels.

## Materials and methods

This study was started after ethical clearance was obtained from the Vinayaka Mission's Sankarachariyar Dental College, Vinayaka Missions Research Foundation (Deemed to be University), Salem, Tamil Nadu, India, with VMSDC/IEC/Approval No: 109, and individual consent was obtained from the diagnosed DM2 patients who visited a dental college for dental problems.

Sample size

The enrolled patients with DM2 visited the outpatient dental department in a dental college and were confirmed to have DM2 by an eminent diabetologist from January 2017 to 2020. Patients were initially screened for HbA1c levels, and those with HbA1c levels greater than 6.5 were confirmed as diagnosed with DM2 and were included in our study. The clinical conditions of DM2, such as oral candidiasis, were confirmed by the presence of discrete curdy white precipitates on the oral mucous membrane; gingivitis was confirmed clinically by signs of gingival inflammation such as erythematous marginal gingiva and absence of gingival recession; periodontitis was confirmed by gingival recession, which indicates loss of attachment; mucormycosis involving hard palate was diagnosed by clinical exposure of necrotic discolored alveolar bone with no history of radiotherapy or bisphosphonate therapy, traumatic dental extraction, or no periapical pathologies such as osteomyelitis from a carious tooth. Xerostomia was clinically identified by ropy saliva and stick sign (mouth mirror sticks to the buccal mucosa during intraoral clinical examination).

Determination of sample size

The sample size was determined by the following formula:

s = Z^2^ * NP (1-P)/d^2 ^(N-1) + Z^2^P (1-P), where N = 60, P = population proportion (assumed to be 50% = 0.5), degree of accuracy (d) = 0.05, margin of error (M) 5% = 0.05, and Z score = 1.960, which refers to the difference from mean standard deviation based on 95% confidence interval.

Inclusion criteria

The study sample included 60 patients who were known to have a diabetic history of more than a year. The study included male and female patients in an equal ratio and only those aged 40-60 years. A complete history of each patient was acquired. The history included parameters such as age, sex, duration of diabetes, family history, any associated illness, and risk factors [6].

Exclusion criteria

Patients with systemic conditions other than DM2 such as burning mouth syndrome, neuropsychiatric illnesses such as depression, glossopyrosis caused by iron, vitamin B12, and folic acid deficiency were excluded. The participants were explained the purpose of the study, and individual voluntary informed consent was obtained. Clinical history and clinical examination data were recorded in a pro forma by oral medicine specialists. The potential benefits of investigations using saliva and peripheral blood were also explained to the patients. The patient was made to take up all the tests with utmost care, and aseptic precautions were ensured, using only sterile diagnostic instruments and laboratory apparatus. Cross-infections involving the doctor and patients were prevented by the use of disposable mouth masks, double gloves, and head caps.

Method of blood and saliva collection

The blood and saliva were collected from each patient by a certified laboratory technician. Briefly, venous blood samples of 5 mL were collected in screw-capped test tubes. The samples are then transferred to the laboratory. The blood samples were allowed to clot, which was carried out at room temperature for approximately 2 h, and the samples were then centrifuged at 3000 rpm for 10 min. This aided in the separation of the serum for the estimation of random blood glucose. This was done following the analyzer method. Then, the patient’s saliva was collected under non-stimulatory conditions. Whole saliva was collected at approximately 1 h after eating, i.e., between 8 a.m and 12 p.m. Patients were asked to spit saliva into a wide-mouthed test tube. Soon after, the saliva was immediately centrifuged at approximately 3000 rpm following the oxidase and peroxidase method. The glucose oxidase (GOX) function is to catalyze the oxidation of β-D-glucose to D-glucono-lactone with the concurrent release of hydrogen peroxide. Hydrogen peroxide (H_2_O_2_) is formed in the presence of peroxidase (POD) when it reacts with p-hydroxybenzoic acid and 4-aminoantipyrine to form a quinoneimine dye complex, which is quantitatively measured by commercially available glucose oxidase-peroxidase (GOD-POD) test kit Liquicheck (AGAPPE Diagnostics Ltd., Kerala). The blood glucose, HbA1c, and saliva glucose values were measured accurately for each patient and then tabulated. The HbA1c value was compared with the random blood glucose value, and a correlation was noted. Subsequently, the blood glucose value was compared with the saliva glucose value, and results revealed that the values were increased or decreased proportionately.

Statistical analysis used

The data were analyzed using IBM Statistical Package for Social Sciences (SPSS) version 22.0 (IBM Corp., Armonk, NY). Descriptive statistics were performed to characterize the sample. The normality tests Kolmogorov-Smirnov and Shapiro-Wilks tests were performed to assess the normal distribution of the data. Descriptive statistics were used to calculate the frequencies, and the Chi‑square test was used to compare the proportions. Cochrane Q test and Pearson’s correlation test were applied to assess the correlation between random blood glucose levels, salivary glucose levels, and HbA1c. The statistical significance was set at 0.05.

## Results

Among 60 study participants, 30 were male, and 30 were female. About 22 (36.6%) were in the age group between 40 and 50 years, and 38 (63.3%) were in the age group between 51 and 60 years. Out of 60, 43 patients have a diabetic history of one to five years, making it a higher frequency with about 72%. The remaining 17 patients have a diabetic history of more than five years, about 28%. Twelve patients show a higher frequency of diabetic history of one year (20%). Patients having a diabetic history of nine years, 12 years, and 15 years show the least frequency of diabetic history (2%). Among the 60 study participants, five (8.3%) had oral candidiasis, eight (13.3%) had dental caries, 11 (18.3%) had gingivitis, one (1.7%) had mucormycosis involving the hard palate, 27 (45%) had periodontitis, and eight (13.3%) had xerostomia. The distribution of oral manifestations among 60 DM cases is shown in Table 1.

**Table 1:** Distribution of oral manifestations in cases of diabetes mellitus

Oral manifestations	Number of cases (N)	Percentage (%)
Candidiasis	5	8.3
Dental caries	8	13.3
Gingivitis	11	18.3
Mucor mycosis on the hard palate	1	1.7
Periodontitis	27	45.0
Xerostomia	8	13.3

The comparison of the mean scores of blood glucose levels between the age groups and gender is shown in Table 2.

**Table 2:** Comparison of the mean scores of blood glucose levels between the age groups and gender *P < 0.05, which is statistically significant.

Variables	Number of cases (N)	Mean ± SD	p-value
Age groups (Years)			
40-50	22	175.86 ± 50.807	0.014*
51-60	38	196.53 ± 51.259	
Gender			
Male	30	192.70 ± 53.834	0.006*
Female	30	185.20 ± 49.995	

The association of the mean scores of durations of DM with oral manifestations of the study participants is shown in Table 3.

**Table 3:** The association of the mean scores of duration of DM with oral manifestations of the study participants DM: Diabetes mellitus.

Oral manifestations	Duration of diabetes mellitus (1-10 years)				
	Mean ± SD	Std. error (S.E)	95% CI (lower-upper)	F	p-value
Candidiasis	7.20 ± 2.168	0.970	4.51-9.89	2.578	0.037*
Dental caries	3.63 ± 2.973	1.051	1.14-6.11		
Gingivitis	3.45 ± 1.916	0.578	2.17-4.74		
Mucor mycosis on the hard palate	10.00 ± 0.000	0.000	0.00-0.00		
Periodontitis	3.93 ± 2.448	0.471	2.96-4.89		
Xerostomia	3.50 ± 4.071	1.439	0.10-6.90		

The correlation of oral manifestations of the study participants with HbA1c values is shown in Table 4.

**Table 4:** Correlation of oral manifestations with HbA1c values *P < 0.05, which is statistically significant.

Oral manifestations	HbA1c, g (%)		Chi-square	p-value
	<7	>7		
Candidiasis	2	3	12.890	0.024*
Dental caries	5	3		
Gingivitis	6	5		
Mucor mycosis on the hard palate	0	1		
Periodontitis	3	24		
Xerostomia	4	4		

The correlation between random blood glucose levels, salivary glucose levels, and HbA1C is shown in Table 5.

**Table 5:** Correlation between random blood glucose levels, salivary glucose levels, and HbA1c *P < 0.05, which is statistically significant.

Variables	Random blood glucose levels (mg/dl)	Salivary glucose levels (mg/dl)	HbA1c g (%)
Random blood glucose Levels (mg/dl)	1.0000	0.935*	0.450*
Salivary glucose levels (mg/dl)	0.935*	1.0000	0.480*
HbA1c g (%)	0.450*	0.480*	1.0000

The association between random blood glucose levels, salivary glucose levels, and HbA1c by Cochrane Q test showed evidence of a statistically significant effect in the dichotomous categorical outcome among study participants (Table 6).

**Table 6:** Association between random blood glucose levels, salivary glucose levels, and HbA1c *P < 0.05, which is statistically significant.

Variables	Fair	Poor	Cochrane Q value	P-value
Random blood glucose level	10	50	84.000	0.000*
Salivary glucose level	60	0		
HbA1c	20	40		

## Discussion

DM is a common systemic disease worldwide. Its prevalence has been increasing. Thus, it must be detected and controlled at an early stage. DM is diagnosed mainly by assessing blood glucose levels. Many commercial devices are available and can accurately measure blood glucose levels. Nevertheless, in all those devices and methods, blood is only taken as the diagnostic body fluid, which is always obtained by an invasive procedure. Thus, the necessity of finding a non-invasive procedure to determine glucose levels without obtaining blood emerged [6]. Accordingly, in this study, the blood and salivary glucose levels of patients with DM2 were measured and compared. The mean HbA1c value was 7 in this study of 60 patients, which was similar to the report by Priya et al., where the mean HbA1c was 7 in their study of 45 patients [3]. In the present study, the Pearson correlation with the random blood glucose, salivary glucose, and HbA1c levels among 60 patients with DM showed a positive correlation. The r-value was 0.912, and the p-value was 0.001**, which indicated significance. This result was similar to that reported by Akasapu et al. [10] who studied 200 patients, compared salivary and blood glucose values, and reported statistically significant results. Similarly, a positive correlation was reported by Azizi et al. who examined 75 patients with DM [9], Kim et al. who analyzed 50 patients with DM [11], and Kumar et al. who examined 50 patients with DM [12]. The survey completed in 2022 by the National Noncommunicable Disease Monitoring Survey revealed a DM incidence of 14.3% in urban areas, which was two times higher than that in rural areas (6.9%) [13].

According to the World Health Organization (WHO), a considerable portion of the population is affected by the multifactorial chronic health condition known as diabetes mellitus (DM), and more adults are predicted to get the disease in the future [14]. The primary biomarker used to evaluate long-term glycemic management in people with diabetes is glycated hemoglobin, or HbA1c, and it is correlated with the emergence of complications [15]. All the observations indicate a significant correlation between salivary glucose and blood glucose levels. The plausible theory is that persistent hyperglycemia causes the production of advanced glycosylation end products, commonly referred to as "Advanced Glycosylation End Products (AGEs)," which cross-link proteins such as collagen and extracellular matrix proteins [16]. This alters basement membrane permeability and makes it more permeabilized to small glucose molecules, resulting in an enhanced leakage of serum-derived components into whole saliva through gingival crevices and sulcular epithelium resulting in a positive correlation of salivary and blood glucose levels [16]. The assessment of salivary glucose can be done at any time as it is readily available. Thus, saliva can be used as an adjunct to blood in measuring glucose levels in patients with DM. Despite the variety of methods of assessing diabetes at an early stage, the assessment of HbA1c level will favor us to have an idea of the mean blood glucose level three months earlier.

Limitations and future scope

The main limitations of this study are that the study sample is unicentric and mainly confined to one particular region of the population from a dental college. The sample size was smaller because only subjects with exclusive type 2 DM were considered, and people with other systemic conditions like glossopyrosis caused by vitamin B12, iron or folic acid deficiency, other comorbidities like hypertension, and underlying depression or other neuropsychiatric disorders that can occur along with type2 diabetes mellitus were excluded. Prediabetics with HbA1c between 4% and 5.6% were not included in the study. The study sample could have been calibrated with other symptoms, such as glossopyrosis, which is not performed in this study. The depth of the periodontal pocket that indicates the severity of periodontitis is not assessed with the WHO Community Periodontal Index (CPI) probe in the study. The mucormycosis involving the hard palate in our study is not confirmed by either culture, biopsy, or histopathological examination by Grocott methenamine silver (GMS) or periodic acid-Schiff (PAS) stain. Salivary flowmetry was not assessed for subjects with xerostomia in our study.

The future scope is aimed at research in artificial intelligence (AI)-based wearable biomedical monitoring devices that use machine learning, and efforts toward its practical application will maximize AI's predictive performance by utilizing large amounts of abundant computational resources and organized data, which will dramatically improve diabetes diagnosis, prevention, and treatment.

## Conclusions

This study proves that as age advances, the glucose levels in the saliva, blood, and HbA1c proportionately increase while there is a simultaneous increase in the diabetic history of all patients. Oral manifestations also worsen with an increase in the duration of diabetes and poor glycemic control as indicated by HbA1c levels. This study provides evidence that the HbA1c level accurately indicates the three-month diabetic state of patients; thus, HbA1c can be an alternative for random blood glucose levels. In addition, this study depicts that salivary glucose levels can very well become a substitute for blood glucose levels. The use of this estimation of salivary glucose levels in practice may avoid the need for invasive procedures for estimating glucose levels in patients with DM.
